# Early DAPT discontinuation after acute myocardial infarction: finding the optimal TARGET

**DOI:** 10.1093/ehjcvp/pvaf075

**Published:** 2025-09-30

**Authors:** Nikolaos Pyrpyris, Kyriakos Dimitriadis, Paolo Calabrò, Felice Gragnano

**Affiliations:** First Department of Cardiology, School of Medicine, National and Kapodistrian University of Athens, Hippokration General Hospital, Athens 11527, Greece; First Department of Cardiology, School of Medicine, National and Kapodistrian University of Athens, Hippokration General Hospital, Athens 11527, Greece; Department of Translational Medical Sciences, University of Campania ‘Luigi Vanvitelli’, Viale Abramo Lincoln 5, Caserta IT- 81100, Italy; Division of Clinical Cardiology, A.O.R.N. ‘Sant'Anna e San Sebastiano’, Caserta 81100, Italy; Department of Translational Medical Sciences, University of Campania ‘Luigi Vanvitelli’, Viale Abramo Lincoln 5, Caserta IT- 81100, Italy; Division of Clinical Cardiology, A.O.R.N. ‘Sant'Anna e San Sebastiano’, Caserta 81100, Italy

The optimal duration of dual antiplatelet therapy (DAPT) after acute myocardial infarction (MI) remains one of the most debated issues in contemporary practice. While current guidelines still endorse 12 months of DAPT as the default strategy,^1^ recent trials, and meta-analyses have consistently shown the benefit of early P2Y_12_ inhibitor monotherapy after 1–3 months of DAPT,^2–4^ therefore supporting this strategy as a favourable alternative.

The TARGET-FIRST trial, presented this year at the European Society of Cardiology Congress, provided further evidence on the safety and efficacy of early transitioning to P2Y_12_ inhibitor monotherapy at 1 month in low-risk patients with acute MI.^5^ Patients who had undergone successful complete revascularization within 7 days after acute MI and had completed 1 month of DAPT with no ischaemic events or major bleeding, were randomized to receive P2Y_12_ inhibitor monotherapy or DAPT for an additional 11 months. A total of 1942 patients were included, with most having one-vessel, single-lesion revascularization. Ticagrelor was the most frequently prescribed P2Y_12_ inhibitor (74%), followed by prasugrel (21%) and clopidogrel (5%). The primary outcome (a composite of all-cause mortality, MI, stent thrombosis, stroke, or Bleeding Academic Research Consortium [BARC] bleeding type 3 or 5) occurred in 2.1% of patients assigned to P2Y_12_ inhibitor monotherapy and 2.2% of those continuing DAPT, meeting noninferiority (difference, −0.09 percentage points; 95% confidence interval [CI], −1.39–1.20; *P* = 0.02 for noninferiority). Clinically relevant bleeding (BARC type 2, 3, or 5) was significantly reduced with P2Y_12_ inhibitor monotherapy (2.6% vs. 5.6%; hazard ratio, 0.46; 95% CI, 0.29–0.75; *P* = 0.002 for superiority). Stent thrombosis was rare and comparable across groups.

Several limitations deserve emphasis. First, the enrolled population was highly selected: patients were relatively low-risk and had tolerated 1 month of DAPT without complications. Second, the trial was underpowered for rare but clinically relevant outcomes, such as stent thrombosis. Third, all patients received a single stent platform, which may limit generalizability.

In conclusion, TARGET-FIRST provides further evidence that early transition to P2Y_12_ inhibitor monotherapy after 1 month of DAPT is feasible in selected post-MI patients. These findings challenge the default application of 12-month DAPT and support the ongoing shift towards flexible, tailored antiplatelet strategies, guided by patient and procedural characteristics.

## Data Availability

As this is a PharmaPulse article, no new data were created.
